# Horizontal and Vertical Transmission of Powassan Virus by the Invasive Asian Longhorned Tick, *Haemaphysalis longicornis*, Under Laboratory Conditions

**DOI:** 10.3389/fcimb.2022.923914

**Published:** 2022-07-01

**Authors:** Wilson R. Raney, Erik J. Herslebs, Ingeborg M. Langohr, Madeline C. Stone, Meghan E. Hermance

**Affiliations:** ^1^Department of Microbiology and Immunology, College of Medicine, University of South Alabama, Mobile, AL, United States; ^2^Department of Pathobiological Sciences, School of Veterinary Medicine, Louisiana State University, Baton Rouge, LA, United States

**Keywords:** Asian longhorned tick, Powassan virus, invasive species, flavivirus, tick-borne disease, vector competence

## Abstract

The Asian longhorned tick, *Haemaphysalis longicornis*, is an ixodid tick native to East Asia that was first detected in North America outside a port of entry in 2017. This invasive species has since been detected in 17 states. As the invasive range of the tick continues to expand, the vector competence of *H. longicornis* for pathogens native to North America must be assessed. Here, we evaluate the vector competence of *H. longicornis* for Powassan virus (POWV) under laboratory conditions. POWV is a North American tick-borne flavivirus that is typically transmitted through the bite of *Ixodes* species ticks. The invasive range of *H. longicornis* is expected to overlap heavily with the geographic range of *Ixodes scapularis* and POWV cases, highlighting the potential for this invasive tick species to amplify POWV transmission in natural foci should the native tick vectors and *H. longicornis* share similar hosts. In these studies, adult female *H. longicornis* ticks were infected with POWV *via* anal pore microinjection. Viral RNA and infectious virions were detected in tick tissues *via* q‐RT‐PCR and focus‐forming assay, respectively. POWV‐injected female ticks were infested on mice, and virus was transmitted to mice during tick feeding, as shown by clinical signs of disease and seroconversion in the tick-exposed mice, as well as the detection of viral RNA in various mouse tissues. A POWV-injected female tick transmitted virus to her larval progeny, indicating that *H. longicornis* can vertically transmit POWV. These naturally-infected larval ticks were also able to transmit POWV to the mouse on which they fed and to the nymphal stage after molting, further demonstrating that *H. longicorni*s can transmit POWV in the horizontal and transstadial modes. Larval and nymphal ticks were also orally infected with POWV while feeding on viremic mice. Additionally, this study provides the first report of POWV neuropathology based on a natural tick transmission model of POWV. Together, our results suggest that the invasive *H. longicornis* tick is a competent vector of POWV. These findings underline the growing danger this tick may pose to human health in the United States. Additional scholarship on the tick’s biology, ecology, and pathogen transmission dynamics in nature will be important towards understanding the full public health impact of this invasive species.

## Introduction

The Asian longhorned tick, *Haemaphysalis longicornis*, is an ixodid tick native to East and Southeast Asia and eastern Russia that has become established in Australia, New Zealand, and several Pacific island nations. This invasive species of tick was first detected in the United States in 2017 on a sheep in rural New Jersey (Rainey et al., 2018). This sheep had not traveled outside of the country, marking this identification as the earliest detection of the tick outside of a U.S. port of entry. Since first being confirmed in the United States, populations of *H. longicornis* have been detected in 17 states along the East Coast and Appalachia, most recently in Georgia in September 2021 (Agriculture, 2021).

*H. longicornis* is an aggressive biter and a host generalist, and in its native range it feeds on mammalian, avian, and human hosts (Hoogstraal et al., 1968). Since its recent establishment in North America, the tick has already been documented feeding on a wide range of species of domestic animals and wildlife, ranging from rodent species to dogs and white-tailed deer (Beard et al., 2018; Agriculture, 2021). The first reported case of *H. longicornis* biting a human in the United States was documented in June 2018 (Wormser et al., 2020), indicating that tick populations are already present in outdoor areas frequented by humans. In its native range, parthenogenetic and bisexual strains of *H. longicornis* have been reported; however, North American populations of the invasive tick appear to be only parthenogenetic (Hoogstraal et al., 1968). The parthenogenetic reproductive ability of *H. longicornis* allows a single female tick to reproduce asexually without fertilization of her embryos. Comparisons of bisexual and parthenogenetic tick populations have suggested that parthenogenetic ticks have a higher dispersal capacity in nature than their bisexual counterparts, and this dispersion can be facilitated by migratory birds (Zhang et al., 2022). *H. longicornis* is adaptable to a wide range of climates across its native and invasive ranges, including seasonal climates. Ecological niche modeling of the potential spread of *H. longicornis* in North America identified several regions beyond the Northeast and East Coast to be suitable for sustaining *H. longicornis* populations, namely the Southeast, the Midwest, the California coast, the Pacific Northwest, and southeastern Canada (Raghavan et al., 2019; Namgyal et al., 2020); large portions of Mexico, Central America, and the Caribbean have been similarly identified (Raghavan et al., 2019). Multiple climate suitability models have confirmed similar potential invasive ranges.

Ticks are important vectors of animal and human pathogens globally. In its native range, *H. longicornis* is a vector of several human pathogens, such as Dabie bandavirus (Family: *Phenuiviridae*, Genus: *Bandavirus*; formerly Severe Fever with Thrombocytopenia Syndrome virus) (Park et al., 2014; Luo et al., 2015; Wang et al., 2015; Zhuang et al., 2018) and *Rickettsia japonica*, the causative agent of Japanese spotted fever (Tabara et al., 2011). Molecular detection of *Anaplasma* species (Qin et al., 2018), *Babesia microti* (Zhang et al., 2017), and tick-borne encephalitis virus (Yun et al., 2016) has also been demonstrated in field-collected *H. longicornis* from China. The diversity of pathogens this tick species can transmit, coupled with its human-biting behavior, underscores the growing and uncertain public health concern *H. longicornis* poses in the United States. A recent laboratory study showed that *H. longicornis* is capable of transovarially transmitting Heartland virus, the North American relative to Dabie bandavirus (Raney et al., 2022). Another study confirmed the presence of viral RNA from Bourbon virus (Family: *Orthomyxoviridae*, Genus: *Thogotovirus*) in field-collected *H. longicornis* in Virginia (Cumbie et al., 2022). The present study assesses the vector competence of *H. longicornis* for the North American tick-borne flavivirus, Powassan virus (POWV; Family: *Flaviviridae*, Genus: *Flavivirus*).

POWV is endemic to the northeastern and midwestern United States, southern Canada, and the Russian Far East. There are two genetic variants of POWV which are serologically indistinguishable: POWV lineage I (POWV I) and POWV lineage II (POWV II, also known as deer tick virus). POWV was first identified as a human pathogen in 1958 following the death of a young boy in Powassan, Ontario, from encephalitis (McLean and Donohue, 1959). The case fatality rate is estimated to be 10% with more than half of survivors facing long-term neurological sequelae (Ebel, 2010). After its initial isolation from the index case, POWV was later confirmed to be maintained in nature between *Ixodes* species ticks and mammalian hosts (McLean and Larke, 1963; McLean et al., 1964). POWV I is maintained in enzootic cycles involving *Ixodes cookei* ticks and groundhogs/mustelids (McLean et al., 1964; McLean et al., 1967) or *Ixodes marxi* ticks and squirrels (McLean and Larke, 1963). POWV II has been isolated from *Ixodes scapularis* ticks and is thought to be maintained in an enzootic transmission cycle between the human-biting *I. scapularis* and white-footed mice (*Peromyscus leucopus*) (Telford et al., 1997; Ebel et al., 2000). Invasive *H. longicornis* have been confirmed parasitizing similar small vertebrate species, namely raccoons, opossums, rabbits, groundhogs, skunks, squirrels, and *Peromyscus* spp. (Agriculture, 2021). Spillover human infections of POWV, some fatal, have been confirmed (Tavakoli et al., 2009) and appear to be increasing in recent years (Hinten et al., 2008; Piantadosi et al., 2016). The duration of time needed for *I. scapularis* ticks to transmit POWV was determined to be as little as 15 minutes post-tick attachment (Ebel and Kramer, 2004), highlighting the risk of virus transmission when humans are bitten by an infected tick that has only briefly fed. Clinical signs of POWV disease in humans include fever and altered mental state progressing to encephalitis and, in the most severe cases, seizures, paralysis, and coma (McLean and Donohue, 1959; Tavakoli et al., 2009; Cavanaugh et al., 2017; Yu et al., 2020).

Human cases of POWV in the U.S. appear to be rising in recent decades (Hermance and Thangamani, 2017; Campbell and Krause, 2020), perhaps due to increased tick and tick-borne disease surveillance or the range expansion of *I. scapularis* populations (Eisen and Eisen, 2018; Campbell and Krause, 2020). Since the first reported POWV case in Ontario (McLean and Donohue, 1959), the geographic range of POWV infections has broadened to include several states in the Northeast, Midwest, and Atlantic seaboard (Campbell and Krause, 2020). This geographic range overlaps significantly with that of counties with confirmed populations of *H. longicornis* (Agriculture, 2021). The introduction of a new tick vector for POWV could amplify established foci of disease and contribute to the proliferation and geographic expansion of the pathogen. Given the uncertainty surrounding the public health implications of such a scenario, it is imperative to understand whether *H. longicornis* ticks are a competent vector of POWV. Here, we demonstrate that *H. longicornis* can transmit POWV II in the horizontal, transovarial, and transstadial modes under laboratory conditions.

## Materials and Methods

### Ethics and Biosafety

All experiments involving virus-infected and mock-infected ticks fed on mice were conducted within a dedicated room of the animal biosafety level 3 (ABSL-3) facility. Infected ticks were handled and housed in arthropod containment level 3 (ACL-3) facilities. Bloodmeals were provided to uninfected ticks for colony maintenance by feeding ticks on guinea pigs in animal biosafety level 2 (ABSL-2) facilities. All biosafety level 3 (BSL-3), ABSL-3, and ACL-3 experiments were performed in accordance with protocols approved by the Institutional Biosafety Committee and the Institutional Animal Care and Use Committee (IACUC). Animal work was conducted using protocols approved by the University of South Alabama (USA) IACUC (protocol numbers 1619216 & 1680702).

### Animals and Ticks

Male and female BALB/c mice were purchased from The Jackson Laboratory (Bar Harbor, ME). Before experimentation, mice were allowed to acclimate to the environment for a minimum of 5 days. Mice were randomly assigned to infection groups and were weighed before and after capsule attachment. During the study, mice were individually housed in ventilated cage systems maintained in a 12:12 light:dark environment. Humidity and temperature were closely regulated for the caging environments. Food and water were provided to mice *ad libitum*.

A pathogen-free colony of parthenogenetic *H. longicornis* ticks was maintained under ACL-2 conditions by feeding ticks on Hartley guinea pigs. This colony originated from parthenogenetic ticks collected in New York state in 2018. This lineage of ticks has been propagated under laboratory conditions for 6 generations without supplementation. Ticks were stored at 21°C at 90-95% relative humidity with a 16:8 light:dark cycle.

### Cells and Virus

A culture of African green monkey kidney (VeroE6) cells was maintained in Dulbecco’s Modified Eagle Medium (DMEM) containing 10% fetal bovine serum (FBS) and 1% penicillin/streptomycin. Cell culture was maintained at 37°C at 5% CO_2_. Deer tick virus (POWV II), Spooner strain, was acquired from the World Reference Center for Emerging Viruses and Arboviruses at the University of Texas Medical Branch. The stock had previously been passaged once on suckling mouse brains and 5 times on Vero cells. It was then passaged once on VeroE6 cells. A focus forming assay (FFA) was used to measure stock virus titers as previously described (Rossi et al., 2012).

### Virus Microinjection and Acquisition by Adult Ticks

Adult female *H. longicornis* ticks were microinjected with POWV II *via* the anal pore as previously described (Talactac et al., 2017). Ticks chosen for microinjection were from the same generation and same time post-molt (i.e. 6 weeks post-molt from nymphal stage to adult). Infection *via* microinjection was achieved by injecting 475 nL of virus stock into the immobilized tick’s anal aperture using glass microneedles, a digitally-operated microinjector with a footswitch, and a dissecting microscope. The volume of virus injected contained approximately 300 focus-forming units (FFU) of POWV II. For the mock-infected groups, an equivalent volume of DMEM media was used. Glass microneedles were made using a micropipette puller (Sutter Instrument) and glass capillaries (World Precision Instruments). The capillaries an internal diameter of 0.530 mm and an outer diameter of 1.14 mm; these micropipettes were pulled such that the tip had a smaller diameter than that of the tick anal pore. Ticks were housed in ACL-3 facilities and were monitored for mortality twice daily for 4 days after microinjection.

#### Detection of Virus in Ticks By Focus-Forming Assay

Whole adult ticks were collected at 14, 21, 28, and 40 days post-injection (d.p.i.) and frozen in media at -80°C. To screen for presence of infectious virus by FFA, whole ticks were individually homogenized in DMEM supplemented with 2% fetal bovine serum, 1% penicillin/streptomycin, and 1% fungizone in a Bead Ruptor 96 Tissue Lyser (OMNI International). Tick homogenate was clarified by centrifugation at 5000 x g at room temperature for 5 minutes. Tick homogenate was cultured on VeroE6 cells in 48-well plates in triplicate. Viral titers in whole adult ticks were determined by FFA as previously described (Rossi et al., 2012).

#### Tick Dissections and RNA Extraction From Tick Tissues

At 14, 21, 28, and 40 d.p.i., adult female ticks from the virus-injected and media-injected cohorts were individually dissected. Salivary glands (SG) and midgut (MG) were harvested from each tick. The remaining tick organs and exoskeleton were collected and designated as carcass (CAR).

SG and MG tick tissues were homogenized in 100 µL TRIzol reagent using a pellet pestle mixer (Thermo Fisher Scientific). Tick CAR samples were homogenized in 1 mL TRIzol reagent with sterile metal beads in a Qiagen TissueLyser II (Qiagen) at 30 hz for 3 minutes. RNA extractions were performed using a hybrid of TRIzol and Qiagen RNeasy Mini Kit protocols. We have previously demonstrated that this hybrid protocol inactivates virus and produces high-quality RNA (Hermance and Thangamani, 2015). Briefly, chloroform was added to tissue homogenate at a ratio of 0.2 mL per 1 mL homogenate. The samples were shaken vigorously for 15 seconds and incubated for 3 minutes at room temperature. Samples were centrifuged at 12,000 x g at 4°C for 15 minutes. The aqueous phase was removed and mixed with an equal volume of 70% ethanol by pipetting. The protocol for the RNeasy Mini Kit was then followed. Total RNA was eluted from the extraction column with 30 µL of nuclease-free water. A Nanodrop 1000 Spectrophotometer (Thermo Fisher Scientific) was used to determine total RNA quantity and purity. Total RNA concentrations of tick tissues ranged from 9.5 to 76.5 ng/µL.

#### Detection of Viral RNA by q-RT-PCR

Absolute quantification of POWV II RNA quantities in tissue samples was determined by quantitative reverse transcription real-time PCR (q-RT-PCR) as previously described (Hermance et al., 2019). Viral RNA quantities are expressed on a Log_10_ scale as the number of POWV II NS5 gene fragment copies per ng of RNA after normalization to a standard curve produced using serial 10-fold dilutions of a 464-base pair POWV II NS5 gene fragment to estimate viral burden. q-RT-PCR was performed using forward (5’ – GATCATGAGAGCGGTGAGTGACT – 3’) and reverse (5’ –GGATCTCACCTTTGCTATGAATTCA – 3’) primers and a probe (/56-FAM/TGAGCACCTTCACAGCCGAGCCAG/36-TAMSp/) specific to POWV II NS5 gene as previously described (Dupuis et al., 2013). When performing the assay, a standard volume of RNA was added to the appropriate wells of a 96-well PCR plate. 10 µM dilutions of the forward and reverse primers and probe were used with reagents from the iTaq Universal SYBR Green One-Step Kit (BioRad). The total reaction volume was 20 µL per well. Plates were sealed and run on a QuantStudio 5 Real-Time PCR System (Thermo Fisher Scientific) at the following cycle settings: 10 minutes at 50°C; 1 minute at 95°C; 10 seconds at 95°C and 30 seconds at 60°C for 45 cycles.

### Horizontal Transmission of Virus From Tick to Mice

For the experiments investigating horizontal transmission of POWV II from ticks to mice, male and female BALB/c mice were 10 weeks old at the time of tick infestation and between 10 -11 weeks old at the start of tick feeding. Tick infestations on mice were carried out using capsules fashioned from 2 mL cryotubes. The base of each tube was cut to leave approximately 3 mm of remaining tube below the screw-cap lid. The tops of the lids were cut to allow for an opening. Capsule bases were attached to a shaved area of the upper dorsum of each mouse using athletic tape and livestock Kamar glue (Kamar Inc., Steamboat Springs, CO). Ticks were placed inside the capsules and a piece of fine mesh fabric was placed under the lid to allow for both tick containment and air exchange. Capsule lids were secured using masking tape. Capsule integrity was checked daily throughout tick infestation. Capsules were reinforced with adhesive and bandages as needed.

During and after tick infestation, mice were monitored twice daily for signs of morbidity and mortality. Body weights and clinical observations were documented once per day. Ticks that did not attach after one week of infestation were removed from the capsules and the corresponding mice were removed from the study. Engorged ticks were retrieved from the capsules after detaching from the skin. Blood was collected from each mouse *via* submandibular bleed at -1, 2 and 5 days post-tick attachment (d.p.a.) under isoflurane anesthesia. Changes in body weight, appearance, neurologic state, behavior, and respiration were documented daily to generate cumulative clinical scores. When mice reached the study endpoint (28 d.p.a.), they were euthanized *via* isoflurane overdose followed by cervical dislocation and terminal cardiac bleed. Euthanasia was administered prior to 28 d.p.a. if mice reached humane endpoints, such as > 20% weight loss, prostrate/unresponsiveness, gasping respiration, or signs of advanced neurologic disease. Necropsies of mock-infected and POWV-infected mice were conducted under ABSL-3 conditions.

The following tissues were harvested from mice during necropsy: brain, liver, kidney, spleen, testes, skin from the tick attachment site, and terminal blood *via* cardiac bleed. Half of each tissue sample was stored in TRIzol Reagent and the other half in 10% neutral-buffered formalin. Formalin was exchanged after 24 hours and was allowed a minimum of 72 hours total contact time with tissues. Terminal blood was equally divided into TRIzol Reagent storage and serum separator tubes. TRIzol-collected tissues were homogenized in a bead beater system as described above. TRIzol-collected samples were screened for viral RNA by q-RT-PCR as described above.

#### Screening Mice for Seroconversion Against POWV

An in-house immunoassay was performed to detect anti-POWV antibodies in the sera of mice infested with POWV-infected ticks. Serum was separated from terminal blood samples taken during necropsy. VeroE6 cells were infected with POWV II at a multiplicity of infection of 0.1 in 48-well plates. After a 1-hour incubation, the virus inoculum was removed and the cells incubated with 2% FBS DMEM media for 2 days. Plates were fixed with 1:1 methanol:acetone for 30 minutes and allowed to airdry. The plates were washed with PBST and blocked with a 5% goat serum solution. Mouse serum was diluted 2-fold from 1:16 to 1:1024. 65 µL mouse serum dilution was added to the plate wells and incubated for 1 hour. The plates were washed with PBST and stained with a secondary goat anti-mouse-HRP antibody for 1 hour. Plates were washed with PBST after staining. 65 µL of AEC developing solution (ImmPACT AEC kit, Vector Labs) was applied to each well to develop the plates according to the product instructions. The plates were wrapped in foil and allowed to develop for up to 30 minutes. Development was stopped by submerging the plates in water. The presence of anti-POWV antibodies were confirmed by identifying the highest dilution of serum where signal (foci) was observed. Additional immunoassays were performed across the dilution range 1:100 to 1:6400 as needed.

#### Histology and RNA *In Situ* Hybridization

After fixation in 10% neutral-buffered formalin, mouse brains were dehydrated with a standard ethanol series and embedded in paraffin. These formalin-fixed paraffin-embedded (FFPE) tissues were sectioned at 5-micron thickness and transferred to glass slides. Prior to staining, slides were baked at 60°C for one hour. Hematoxylin and eosin (H&E) staining was performed according to routine procedures.

RNA *in situ* hybridization (RNA ISH) was performed using an RNAscope 2.5 HD Duplex Detection Kit (Chromogenic) (Advanced Cell Diagnostics) according to the kit instructions. This system hybridizes RNA-specific probes to an RNA target and then binds them to a cascade of signal amplification molecules. The target probe is bound to a horseradish peroxidase (HRP)-conjugated label probe or an alkaline phosphatase (AP)-conjugated label probe. The signal is detected following the addition of the chromogenic substrate. Briefly, tissue sections were deparaffinized and endogenous peroxidases were quenched with hydrogen peroxide for 10 minutes. The slides were gently boiled with RNAscope Target Retrieval Solution for 15 minutes and incubated with RNAscope Protease Plus reagent for 30 minutes at 40 °C. Brain sections then underwent probe hybridization with a POWV positive-sense RNA probe (catalog number 415641-C1) to detect POWV genomic RNA and a *Mus musculus* GFAP probe (catalog number 313211-C2). The POWV RNAscope probe is cross-reactive with POWV II RNA and thus does not distinguish between POWV Lineage I and Lineage II. Signal amplification and detection were then performed following the kit protocol for FFPE tissues. Slides were counterstained with a 50% solution of Gill’s hematoxylin I. H&E and RNAscope-stained sagittal brain sections were examined by a board-certified veterinary pathologist.

### Vertical Transmission of POWV

Engorged ticks were individually housed in the ACL-3 after feeding on mice to facilitate oviposition. At 14 days (± 1 day) post-oviposition, 3 pools of 50 eggs were collected from each egg mass. After the remaining eggs hatched, 4 pools of 50 larvae were collected. Egg and larval pools were homogenized in TRIzol reagent, and RNA was extracted from each homogenate as described above. Egg and larval pools were screened for viral RNA by q-RT-PCR as described above. Fed female carcasses were similarly homogenized in TRIzol reagent, extracted of RNA, and screened by q-RT-PCR. Additional larval pools of 150 larvae each were taken from egg clutches for titration of POWV by FFA. Larval pools were homogenized, clarified, and cultured on VeroE6 cells as described above.

### Acquisition of POWV by Ticks Feeding on Viremic Mice

For the experiments involving pathogen-free *H. longicornis* larvae and nymphs fed on viremic mice, male and female BALB/c mice were 7 weeks old at the time of tick infestation. Mice were intraperitoneally injected with 10^4^ FFU of POWV II and infested with pathogen-free *H. longicornis* larvae or nymphs on the same day using the capsule feeding method described above. Up to 50 *H. longicornis* larvae or 15 nymphs were added to each mouse capsule. Blood was collected from each mouse *via* submandibular bleed at 1- and 3-days post-infection. Mouse blood samples were screened for viral RNA by q-RT-PCR as described above. Engorged ticks were retrieved from the capsules after detaching from the skin. Once all ticks were collected, mice were euthanized and necropsies were performed as described above. Additional mice were mock-infected by intraperitoneal injection with DMEM media. Pathogen-free *H. longicornis* larvae and nymphs were fed on the mock-infected mice.

Fed ticks were transferred to the ACL-3. Pools of 5 fed larvae and 3 fed nymphs were collected and homogenized in TRIzol. Fed larvae and nymph pools were screened for viral RNA by q-RT-PCR as described above. Additional fed larvae (n = 5 per pool) were collected in DMEM media supplemented with 2% fetal bovine serum, 1% penicillin/streptomycin, and 1% fungizone. The pooled fed larvae were homogenized in media, and the clarified tick homogenate was screened for the presence of infectious virus by FFA as described above. The remaining tick homogenate from each pooled larvae sample was cultured on VeroE6 cells for 10 days. At 10 days post-infection, cell supernatant was harvested and screened for viral RNA by q-RT-PCR as described above.

### Statistical Analysis

Statistical analyses were performed with GraphPad Prism 9.3.1 software. Statistical significance was determined for normally-distributed datasets using a one-way ANOVA followed by Tukey’s multiple comparisons test. P-values less than 0.05 and 0.01 were regarded as significant and highly significant, respectively.

## Results

### POWV II Infection in Anal Pore Microinjected Ticks

**Figure 1:**
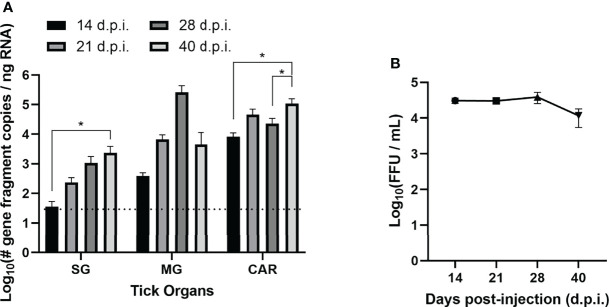
Acquisition and maintenance of POWV by *H. longicornis* ticks *via* anal pore microinjection. **(A)** Detection of Powassan virus (POWV) RNA by real-time quantitative reverse transcription polymerase chain reaction (q-RT-PCR) of POWV-injected *H. longicornis*. Microinjected adult female ticks were dissected at 14, 21, 28, and 40 days post-injection (d.p.i.). Tick organs were screened individually: salivary glands (SG), midgut (MG), and carcass (CAR). Viral RNA quantities are expressed as the number of NS5 gene fragment copies per ng of RNA after normalization to a standard curve. The data are presented as means with error bars representing standard error of the means. Statistical significance was determined using one-way ANOVA followed by Tukey’s multiple comparisons test. Limit of detection ~31 gene fragment copies per ng RNA. *p < 0.05. **(B)** Detection of infectious POWV virions in whole microinjected ticks by focus-forming assay (FFA). Microinjected adult female ticks were collected at 14, 21, 28, and 40 d.p.i., and whole ticks were homogenized in DMEM media. Tick homogenate was cultured on Vero E6 cells, and viral titers were determined by FFA. The data are represented as the focus-forming units (FFU) per mL of POWV for each time point.

49 adult female *H. longicornis* were selected from a pathogen-free colony and divided into virus and control cohorts. 34 ticks were microinjected in the anal pore with 300 FFU of POWV II. The remaining 15 ticks were microinjected in the anal pore with an equivalent volume of DMEM media. At time points ranging from 14 to 40 days post-injection (d.p.i.), ticks were dissected and the following samples were harvested: salivary gland (SG), midgut (MG), and carcass (CAR). Detection of POWV II RNA in the tick samples was demonstrated by q-RT-PCR (**Figure 1A** and **Table 1**). For the POWV II-injected cohort of ticks, viral RNA levels followed a general upward trend over time. Viral RNA was highest at 40 d.p.i. for SG tissue and was significantly higher than at 14 d.p.i. (p<0.05). Viral RNA peaked at 28 d.p.i. in MG tissue; however, this increase was not statistically significant compared to other time points. In the CAR tissue, viral RNA was significantly higher at 40 d.p.i. than at 14 d.p.i. (p<0.05) and 28 d.p.i. (p<0.05). As expected, no tick tissues from the media-injected cohort screened positive for POWV II RNA.

**Table 1:** Rate of detection of POWV RNA by q-RT-PCR and infectious POWV by FFA in adult female *H. longicornis* at 14, 21, 28, and 40 days post-injection (d.p.i.).

Treatment	q-RT-PCR* detection of POWV RNA (number positive/number tested)			FFA titration of POWV
	Salivary glands (SG)	Midgut (MG)	Carcass (CAR)	Whole tick
Media-inj.	0/15 (0%)	0/15 (0%)	0/15 (0%)	0/12 (0%)
POWV-inj. (14 d.p.i.)	3/5 (60%)	5/5 (100%)	5/5 (100%)	4/4 (100%)
POWV-inj. (21 d.p.i.)	4/5 (80%)	5/5 (100%)	5/5 (100%)	4/4 (100%)
POWV-inj. (28 d.p.i.)	5/5 (100%)	5/5 (100%)	5/5 (100%)	4/4 (100%)
POWV-inj. (40 d.p.i.)	3/3 (100%)	3/3 (100%)	3/3 (100%)	4/4 (100%)

*Real-time quantitative reverse transcription polymerase chain reaction.

POWV II-injected ticks and media-injected ticks were titered for infectious POWV by a FFA (**Figure 1B** and **Table 1**). Foci were produced by all POWV II-inj. ticks at each time point, indicating that infectious virions were present in the tick body at every time point. The titer of infectious virus showed no significant change from 14 d.p.i. to 40 d.p.i. Media-inj. ticks did not produce any foci, which supported the negative q-RT-PCR results indicating the media-inj. ticks were negative for POWV II.

### Horizonal Transmission of POWV II From Ticks to Mice

36 additional adult female *H. longicornis* ticks were selected and their ability to horizontally transmit POWV II to BALB/c mice was assessed. 7 ticks were microinjected with DMEM media and the remaining 29 with POWV II. Mice were singly-housed and exposed to an individual tick at 28 d.p.i. Each tick was contained within a feeding capsule affixed to the mouse dorsum. Ticks were monitored daily for position, attachment, and engorgement. Four of the media-inj. ticks and 7 of the POWV II-inj. ticks engorged and fed to repletion. Two additional POWV II-inj. ticks, which fed on Mouse 4 and Mouse 6, attached but only partially fed. The tick that fed on Mouse 4 attached, fed, then relocated itself to a new feeding site 3 different times over the course of 8 days. At 8 days post-attachment (d.p.a.), this tick was partially engorged but was removed from the mouse because the capsule was damaged beyond repair. The tick that partially fed on Mouse 6, stopped engorging at 6 d.p.a. and was removed from the mouse skin at 8 d.p.a. Both partially-fed ticks were carefully removed from the mouse skin using fine forceps. All engorged and partially-fed ticks were collected and individually housed in the ACL-3 laboratory to facilitate oviposition.

**Figure 2:**
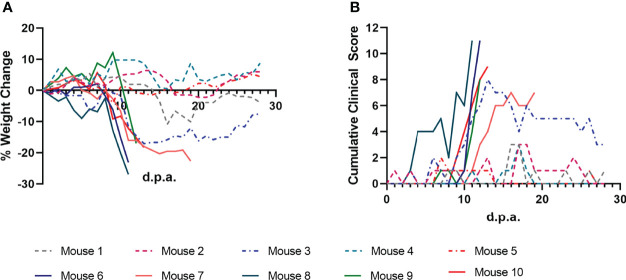
Clinical markers of POWV disease progression in mice infested with POWV-infected *H. longicornis*. Mice 1 - 9 were each infested with an individual POWV II-injected adult female tick. Mouse 10 was infested with larvae (derived from the adult female tick fed on Mouse 6). **(A)** Percent weight change is represented as a line plot relative to the weight at study day 0. Weight loss greater than 20% was used as a humane endpoint for euthanasia. **(B)** Clinical disease scores are represented as a line plot with higher cumulative scores representing more severe disease. Cumulative clinical scores were assigned to each mouse daily based on weight loss, appearance, neurological signs of disease, and behavior. Lines that end before 28 days post-attachment (d.p.a.) indicate criteria for euthanasia were reached for a certain mouse.

Mice were monitored daily during tick infestation and post-tick removal for clinical signs of POWV disease. Every day, each mouse was weighed and assigned a cumulative clinical score based on specific criteria: weight loss, appearance, respiration, behavior, and neurologic changes (**Figure 2**). Mice that met any predefined humane endpoints were immediately euthanized, while surviving mice were euthanized at the study endpoint (28 d.p.a.). All nine mice that were fed upon by POWV II-inj. adult ticks displayed clinical signs of disease; however, the mice that survived to 28 d.p.a. only exhibited signs of mild, febrile illness (e.g., ruffled fur, reduced grooming, lethargy, etc.). Four mice (Mice 6 - 9) displayed signs of severe illness and reached humane endpoints that warranted euthanasia before 28 d.p.a. (**Figure 2** and **Table 2**). These 4 mice displayed dramatic weight loss (**Figure 2A**) and sharp increases in clinical disease scores (**Figure 2B**), whereas the remaining mice that survived to the study endpoint consistently had only mildly fluctuating clinical scores and % weight loss. As expected, no mice that were fed upon by media-inj. ticks displayed signs of disease (**Supplementary Figure 1**).

**Table 2:** Disease in mice infested with POWV-injected adult female *H. longicornis*.

Treatment	Mortality (Number Succumbed/Total)	Mild to Severe Clinical Signs (Number with Clinical Signs/Total)	Viral RNA Present in Brain (Number Positive/Total)	Seroconversion (Number Seroconverted/Total)
**Media-injected female ticks**	0/4 (0%)	0/4 (0%)	0/4 (0%)	0/4 (0%)
**POWV-injected female ticks**	4/9 (44.4%)	9/9 (100%)	8/9 (88.9%)	8/8 (100%)*

*No serum was taken from one mouse which was found moribund at the time of necropsy.

**Figure 3:**
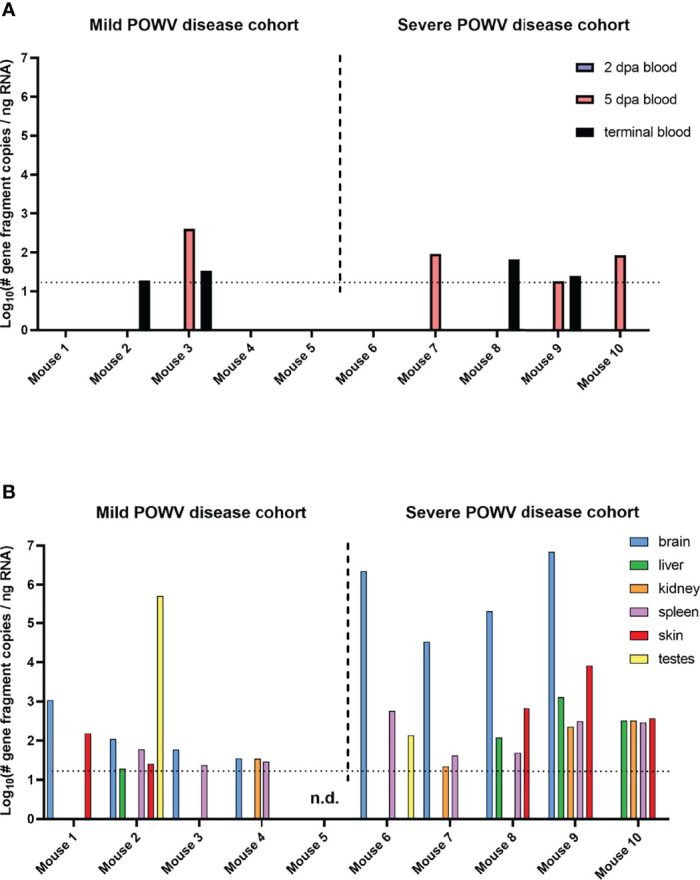
Detection of POWV RNA in blood **(A)** and tissues **(B)** collected from mice infested with POWV-infected *H. longicornis*. Mice 1 - 9 were each infested with an individual POWV II-injected adult female tick. Mouse 10 was infested with larvae (derived from the adult female tick fed on Mouse 6). Data are shown for mice with mild disease that survived to the study endpoint of 28 days post-attachment (d.p.a.) and those with severe disease that qualified for euthanasia before 28 d.p.a. Blood samples were taken at 2 d.p.a., 5 d.p.a., and at the time of necropsy. POWV RNA was detected in samples *via* q-RT-PCR. Viral RNA quantities are expressed as the number of NS5 gene fragment copies per ng of RNA after normalization to a standard curve. Each bar represents a single data point from a single tissue. Limit of detection ~15 gene fragment copies per ng RNA. n.d., not detected.

Blood and tissue samples collected from mice fed upon by media-inj. and POWV II-inj. adult ticks were screened for POWV II RNA by q-RT-PCR (**Figure 3**). No POWV II RNA was detected in tissues from mice exposed to media-inj. ticks; however, POWV II RNA was detected in every type of tissue collected from mice exposed to POWV-inj. ticks. The highest levels of POWV II RNA were found among the brains of mice that exhibited severe disease post-tick removal (Mice 6 - 9). The average quantities of POWV II RNA in brain tissues from the mild disease and severe disease cohorts were 2.10 and 5.75 Log_10_(# gene fragment copies/ng RNA), respectively. There was little difference in POWV II RNA levels between the severe disease group and the mild disease group for the tissues collected other than brain. One noteworthy exception is the testes collected from Mouse 2 from the mild disease group which had a level of viral RNA as high as that of the brains from mice that displayed severe POWV disease.

Terminal blood samples were used to determine if the mice seroconverted relative to POWV. Serum was separated from each blood sample, and an immunoassay using POWV II-infected VeroE6 cells as antigens was performed with dilutions of each serum sample to detect POWV-specific antibodies. All 8 of the mice fed upon by POWV II-inj. adult ticks whose serum was screened had detectable POWV-specific antibodies (**Table 2**). Antibodies were detected as far as a 1:6400 dilution of serum. Even Mouse 5, which had no detectable POWV II RNA in blood or terminal tissues, screened positive for POWV-specific antibodies. As expected, none of the mice fed upon by media-inj. ticks seroconverted relative to POWV.

#### Neuropathology in Mice Infested With POWV II-Injected *H. longicornis* Adult Ticks

**Figure 4:**
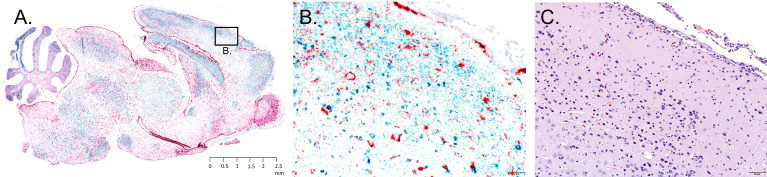
Representative neuropathology in a mouse infested with POWV-infected *H. longicornis*. Mouse 6 was infested with an individual POWV II-injected adult female tick. **(A)** RNA *in situ* hybridization detection of POWV RNA (blue-green signal) and *Mus musculus* GFAP RNA (red signal) in the whole brain sagittal cross-section. Section was counterstained with hematoxylin. Scale bar = 2.5 mm. **(B)** Inset image of RNA *in situ* hybridization detection of POWV RNA and *Mus musculus* GFAP RNA in the cerebral cortex. Scale bar = 50 microns. **(C)** H&E stained cerebral cortex. Scale bar = 50 microns.

Sagittal brain sections were harvested from each mouse fed upon by media-inj. and POWV II-inj. adult female *H. longicornis* ticks. A section from each brain sample underwent H&E staining, while a serial section underwent RNA ISH staining. Histologic alterations in the brain were most severe and widespread in the mice fed upon by POWV II-inj. ticks that succumbed at 11 and 12 d.p.a. (Mice 6, 8, and 9), with primary involvement of the cerebral cortex, hippocampus (mainly CA1, CA2 and dentate gyrus regions), olfactory bulb (mainly the granular and mitral layers), and midbrain (including thalamus and hypothalamus regions) (**Figure 4C**). The histologic findings consisted of shrunken, angular, hypereosinophilic and pyknotic (necrotic) neurons and karyorrhectic glial cells, accompanied by patchy gliosis and minimal to mild lymphocytic infiltrate of the leptomeninges and perivascular spaces as well as occasional spongiosis. In Mouse 7, which succumbed at 20 d.p.a., and in Mice 1 - 5, which survived to the study endpoint, alterations were restricted to occasional patchy gliosis in the cerebral cortex, hippocampus, and midbrain as well as minimal to mild leptomeningeal and occasional perivascular inflammatory infiltrate.

The neuronal morphologic changes in the mice that succumbed at 11 and 12 d.p.a. (Mice 6, 8, and 9) correlated with detection of positive-sense genomic POWV RNA *via* RNA ISH staining. POWV RNA was observed within neuronal cell bodies and processes (**Figures 4A, B**). In contrast to the histologically evident alterations, POWV RNA had a more widespread distribution throughout the brain in these mice, including in the cerebral cortex and nuclei (striatum), hippocampus, anterior olfactory nuclei with extension into the olfactory bulb, brainstem (thalamus, pons, medulla oblongata), and cerebellum (mostly in the granular cell layer, with fewer individual POWV labeled cells in the molecular layer and cerebellar nuclei). This was variably accompanied by astrogliosis, highlighted by RNA ISH detection of *Mus musculus* GFAP RNA (**Figures 4A, B**).

Mice that survived until 20 to 28 d.p.a. (Mice 1 - 5 and Mouse 7; all fed upon by POWV II-inj. ticks) had variable, often only mild, patchy astrogliosis, indicated by GFAP RNA detection. Astrogliosis in these mice was mostly detected in the cerebellum, midbrain, deep aspect of the cerebral cortex, and hippocampus. This astrogliosis was occasionally associated with viral RNA in individual neurons. There were no readily apparent alterations or detectable viral RNA in the brains of the mice fed upon by media-inj. ticks (Mice 11-14).

### Vertical Transmission of POWV II

Microinjected fed female ticks were individually housed to facilitate oviposition. Once oviposition was complete, the female tick carcasses were removed and screened for POWV II RNA by q-RT-PCR (**Table 3**). Eight of the 9 POWV II-inj. fed female tick carcasses reported positive for POWV II RNA, demonstrating that POWV was present in the tick bodies for both the mild disease cohort and severe disease cohort of mice. Detectable viral RNA dropped below the limit of detection for the ninth POWV II-inj. fed female tick carcass which did not test positive for POWV RNA; however, this tick was clearly infected with POWV at the time of feeding as it was capable of transmitting virus to Mouse 7. As expected, none of the media-inj. fed female tick carcasses tested positive for POWV II RNA.

**Table 3:** Detection of POWV RNA by q-RT-PCR and infectious POWV by FFA in *H. longicornis* fed adult carcasses, eggs, and larvae.

Treatment	q-RT-PCR detection of POWV RNA (number positive / number tested)			FFA titration of POWV
	Fed female carcass	Egg pools	Larval pools	Larval pools
Media-inj.	0/4 (0%)	0/12 (0%)	0/16 (0%)	0/4 (0%)
POWV-inj.	8/9 (88.9%)	0/21 (0%)*	2/30 (6.7%)	0/9 (0%)*

*No pools were collected for the lineage of ticks derived from the female tick fed on Mouse 6 whose larvae screened positive for POWV RNA.

Transovarial transmission was first measured by detecting POWV II RNA by q-RT-PCR in pools of tick eggs derived from the microinjected adult female ticks (**Table 3**). Before hatching, 3 pools of 50 eggs per egg clutch were removed and screened for viral RNA. This analysis was repeated by removing and screening 4 pools of 50 larvae from each clutch after hatching was complete. An exception to this analysis was the progeny from the adult female tick that only fed partially on Mouse 6. This tick produced only a small egg mass during oviposition. Since Mouse 6 displayed severe clinical signs of POWV disease and eventually succumbed, egg samples were not initially collected to preserve enough immature ticks of this lineage for further analysis. However, pools of larvae derived from the female tick that partially fed on Mouse 6 were eventually collected and screened positive for POWV II RNA *via* q-RT-PCR analysis (**Table 3**). Aside from the larvae derived from the female tick that partially fed on Mouse 6, viral RNA was not detected in any other egg pools or larval pools derived from the other microinjected fed female ticks. These negative results were supported by the FFA screening of larval pools where none of the larval pools reported positive for infectious POWV (**Table 3**). The larvae derived from the Mouse 6 lineage were not screened by FFA due to the small egg clutch size.

The lineage of ticks derived from the adult female tick that partially fed on Mouse 6 followed a pattern unique from the other tick lineages that arose from this study. After confirming the presence of POWV II RNA in the brain of Mouse 6, the subsequent larvae from the mother tick were considered to be putatively-infected, which was ultimately confirmed by q-RT-PCR analysis (described above) in which these larvae reported positive for POWV II RNA. Therefore, these larvae were fed on an additional mouse, Mouse 10, which displayed signs of POWV disease after larvae attachment and was euthanized at 13 d.p.a. (**Figures 2**, **3**). Although sample size limitations precluded the FFA screening of larvae derived from the Mouse 6 lineage, the clinical outcome of the mouse on which these larvae fed (Mouse 10) demonstrates that infectious POWV was indeed present in these larvae. These fed larvae and post-molt nymphs were screened for viral RNA by q-RT-PCR. 2/3 (67%) of fed larval pools and 1/2 (50%) of nymph pools were positive for POWV II RNA, demonstrating transstadial transmission of POWV in the lineage of ticks derived from Mouse 6.

### Acquisition of POWV II by Tick Feeding

The majority of this study made use of adult ticks artificially infected by POWV II microinjection through the anal pore. However, to demonstrate tick acquisition of POWV *via* the natural route of feeding on a viremic host, additional mice and pathogen-free immature *H. longicornis* ticks were selected. Pathogen-free larval and nymphal ticks were fed to repletion on mice that were intraperitoneally injected with POWV II. Non-terminal blood samples were collected from the mice at 1- and 3-days post-injection, and the blood samples were positive for POWV II RNA (**Supplementary Figure 2**).

**Figure 5:**
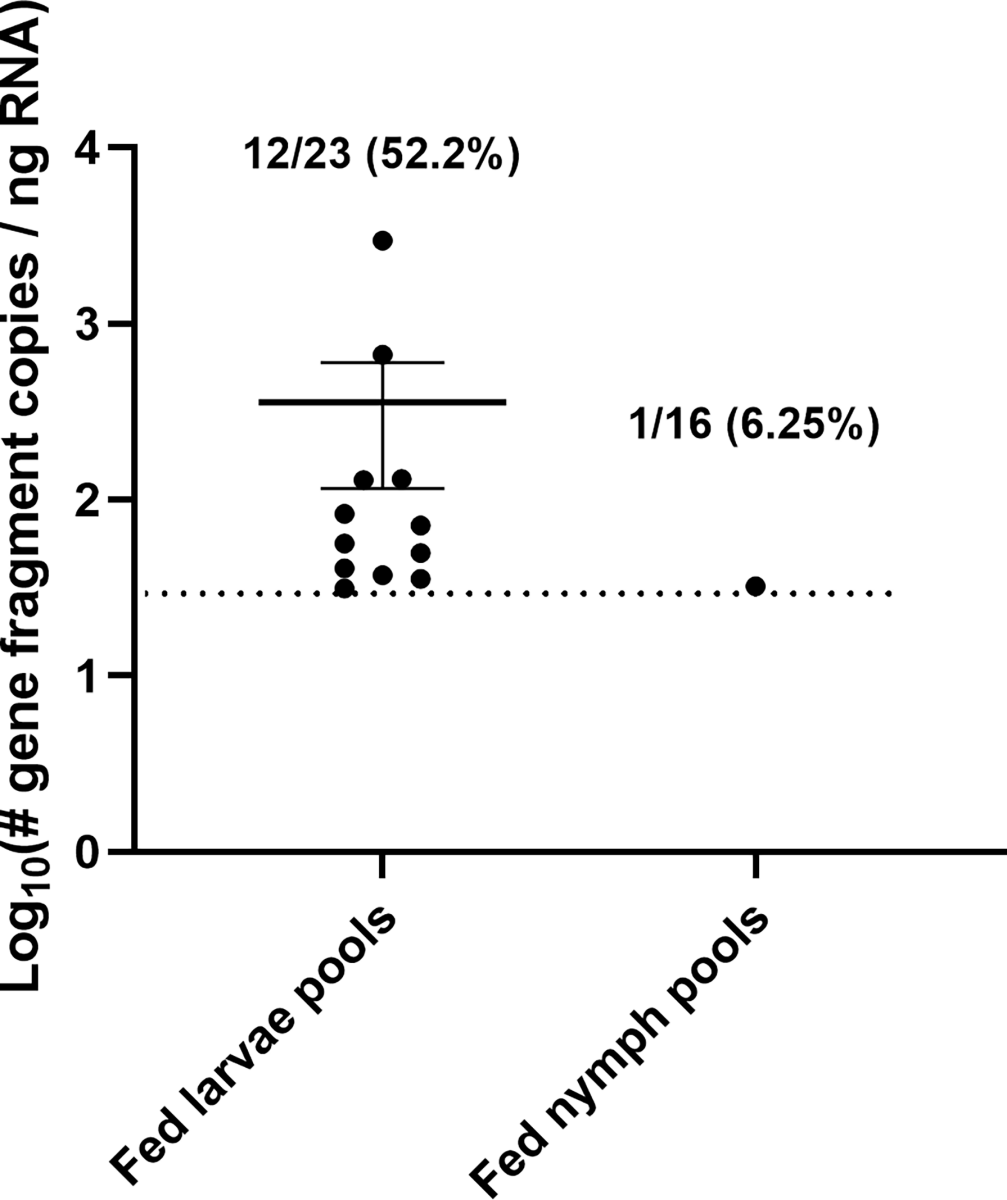
Acquisition of POWV by *H. longicornis* ticks fed on viremic mice. Detection of POWV RNA in engorged larvae and nymphs that fed on POWV-infected mice. Ticks were pooled at n=5 for larvae and n=3 for nymphs. POWV RNA was detected in pooled fed tick samples *via* q-RT-PCR. Viral RNA quantities are expressed as the number of NS5 gene fragment copies per ng of RNA after normalization to a standard curve. The data are presented as means with error bars representing standard error of the means. Limit of detection ~31 gene fragment copies per ng RNA.

Fed larvae were collected in pools of 5 and fed nymphs in pools of 3 for quantifying viral RNA. Pools of fed ticks were homogenized and screened for POWV II RNA by q-RT-PCR. Viral RNA was detected in 12/23 fed larval pools (52.2%) and in 1/16 fed nymph pools (6.25%) (**Figure 5**). In an attempt to screen for infectious POWV in fed tick larvae, additional fed larval pools were homogenized in media and assayed by FFA (n = 5 fed larvae per pool). True infectious foci were difficult to distinguish from tick debris in these FFAs, so an aliquot of each fed larval pool was cultured on VeroE6 cells. At 10 days post-infection, cell supernatant samples were harvested and POWV II RNA was detected by q-RT-PCR in 11/12 (91.7%) supernatant samples. As expected, none of the fed larvae and nymphs exposed to mock-infected mice (a.k.a., mice intraperitoneally injected with DMEM media) screened positive for viral RNA or infectious POWV.

## Discussion

Our data suggest that parthenogenetic *H. longicornis* can serve as vectors of POWV lineage II, also known as deer tick virus. Using adult *H. longicornis* that were microinjected with POWV II, this study demonstrates acquisition and maintenance of virus in adult ticks, horizontal transmission of virus from adult *H. longicornis* to BALB/c mice, and vertical transmission of virus from a female tick to her progeny.

Anal pore microinjection is an established procedure that delivers specific amounts of virus directly into the tick alimentary canal (Kariu et al., 2011; Talactac et al., 2017; Talactac et al., 2018), which is the first organ system that virus contacts in ticks that naturally acquire virus during feeding. Here, anal pore microinjection was shown to be a suitable method for artificially infecting adult *H. longicornis* ticks with POWV. We further demonstrated the tick’s ability to acquire virus through a natural route: pathogen-free immature ticks feeding on a viremic host. Though these ticks were screened for virus before molting, the detection of viral RNA in fed ticks implies that virus was introduced into the tick midgut during blood feeding. Infection of midgut tissue is the first step in pathogen acquisition in ticks. Through anal pore microinjection of adult ticks and feeding immature ticks on viremic mice, the data demonstrate the ability of *H. longicornis* to acquire virus at all life stages through contact of POWV with midgut tissue, whether infected naturally or artificially. The microinjected ticks harbored infectious virus out to the 40 d.p.i. time point, as demonstrated by FFA and supported by increasing levels of viral RNA over the same time period. Since *H. longicornis* is a three-host tick, pathogen transmission can be dependent on the time needed for an individual tick to locate its next host. Since these ticks can maintain infectious virus for at least several weeks under laboratory conditions, we would expect this viral maintenance to facilitate tick-to-host viral transmission in nature. In the present study, POWV II RNA was detected in tick salivary glands, midgut, and carcass until the latest time point, 40 d.p.i. The POWV II RNA levels in the salivary glands and carcass followed a general upward trend over time, suggesting that an active POWV infection occurred during this interval in the POWV-injected ticks.

Data from the present study show that adult female *H. longicornis* microinjected with POWV II were able to horizontally transmit POWV to BALB/c mice. All mice fed upon by POWV II-inj. adult ticks displayed clinical signs of disease with 4 out of 9 mice displaying signs of severe POWV disease and eventually reaching criteria for euthanasia. The mice that showed severe signs of disease had a higher average quantity of POWV II RNA in their brains than in the surviving mice that displayed only mild clinical signs of disease. These results align with the expected disease pattern and clinical outcome of POWV-infected BALB/c mice, which have previously been confirmed as a suitable animal model for POWV lineages I & II infection and pathogenesis (Holbrook et al., 2005; Hermance and Thangamani, 2015; Hermance et al., 2020; Santos et al., 2021). Several previous studies have shown that BALB/c mice needle inoculated with POWV I or POWV II display signs of disease that mimic the course of human infection in which neurological manifestations are preceded by generalized febrile illness (Holbrook et al., 2005; Hermance and Thangamani, 2015; Hermance et al., 2020; Santos et al., 2021). However, *in vivo* infection models based on needle inoculation of a tick-borne virus do not accurately represent the natural route of virus transmission whereby an infected tick transmits virus to the vertebrate host on which it feeds. Tick feeding is a complex process known to be facilitated by a cocktail of bioactive factors in tick saliva, which can modulate host wound healing, pain and itch responses, hemostasis, and the innate and adaptive immune response (Ribeiro et al., 2006; Kazimirova et al., 2017; Wikel, 2018). While previous studies have employed the natural route of POWV transmission (i.e., tick feeding) to examine skin histopathology and the host’s early cutaneous immune response to POWV infection at the tick feeding site (Hermance and Thangamani, 2014; Hermance et al., 2016), no study has used a tick transmission model to examine the histopathological changes in response to disseminated POWV infection. Here, neuropathology was assessed in the brains of BALB/c mice exposed to POWV II-inj. adult female *H. longicornis* ticks. Neuronal necrosis, karyorrhectic glial cells, and perivascular inflammation were associated with extensive labeling of POWV RNA and with astrogliosis in the brains of tick-exposed mice that succumbed to disease at 11 and 12 d.p.a. For mice that survived to the study endpoint (28 d.p.a.), mild, often patchy, astrogliosis was occasionally associated with viral RNA in individual neurons. Although neuropathological analysis was not the focus of this study but was included as additional data that supports horizontal transmission of POWV II by *H. longicornis ticks*, we felt it was important to report these data since no other study has used a natural tick transmission model to characterize POWV neuropathology.

One of the adult female *H. longicornis* ticks in the present study was able to transmit POWV II vertically. Although this was confirmed by the presence of POWV II RNA in that tick’s progeny (larvae and nymphs), and by the transmission of POWV from the larval progeny to Mouse 10, an estimate of the rate of vertical transmission is unknown due to sample size limitations. *H. longicornis* appears as two strains: a bisexual strain and a parthenogenetic strain (Hoogstraal et al., 1968). Reports of the tick in the United States have been confined to the latter strain, meaning a single adult female tick can lay hundreds of eggs without mating. Evidence that this invasive tick species can transmit POWV in the transovarial mode without the need for a mate is worrying from a public health standpoint; however, the full extent of this threat can only be understood by future work investigating the rate of transovarial and transstadial POWV transmission (namely larvae-to-nymph and nymph-to-adult). Such studies should employ larger cohorts of ticks than demonstrated here since vertical transmission often only occurs with low positivity rates.

The scope of this dataset is limited by the artificial method of adult tick infection and the sample size of certain tick cohorts. Anal pore microinjection is not a natural route of virus acquisition by ticks, nor does it account for transstadial survival of virus following the acquisition of an infectious bloodmeal by immature ticks. In the present study, larvae derived from a microinjected female tick harbored POWV RNA, transmitted POWV to a mouse host during feeding, and maintained viral RNA after molting to nymphs; however, the original infection of this tick lineage was of the artificial method rather than the natural oral route. Additionally, this study did not investigate the maintenance of POWV from the nymphal stage to adulthood, transstadial survival of POWV following infection *via* the oral route, or subsequent horizontal transmission of POWV following oral acquisition and molting. The low viral positivity rates seen in the assays involving immature ticks could be better understood in studies employing larger cohort sizes.

Ongoing reports of confirmed *H. longicornis* populations in the United States have demonstrated both a rapid spread of the tick and a diverse range of hosts. Among the reported hosts for the invasive tick are several small and medium-sized vertebrates that participate in enzootic cycles of POWV I and POWV II with *Ixodes* spp. ticks. These hosts include *Peromyscus* spp., groundhogs (woodchucks), skunks, and squirrels. (Ebel et al., 2000; Ebel, 2010; Agriculture, 2021). Furthermore, white-tailed deer are heavily parasitized by *I. scapularis*, the main vector of POWV II, and there have been multiple reports of *H. longicornis* collected from white-tailed deer in North America (Agriculture, 2021). In Staten Island, New York, all three life stages of *H. longicornis* were collected from white-tailed deer where they were found co-feeding alongside two native tick species, *I. scapularis* and *Amblyomma americanum* (Tufts et al., 2019). These findings raise the possibility of *H. longicornis* ticks acquiring pathogens from other tick species *via* co-feeding on the same vertebrate host. Future studies should investigate the potential for non-viremic co-feeding transmission of POWV between POWV-infected ixodid ticks and naïve *H. longicornis* co-feeding on the same host. The present study demonstrated that naïve *H. longicornis* can acquire POWV by feeding on viremic hosts. This finding captures one possibility of how the invasive tick may intersect existing sylvatic cycles of POWV through its reported affinity for established vertebrate POWV reservoirs.

*H. longicornis* poses a substantial public health threat to the United States. Because the tick was only first confirmed as an invasive species in 2017, little is known about the tick’s ability to maintain and transmit North American pathogens. Human bites have also been recorded (Wormser et al., 2020). The geographic range of human POWV cases and the invasive range of *H. longicornis* already overlap heavily in the Northeast. POWV cases are also prevalent in the Midwest (Campbell and Krause, 2020), a region where *H. longicornis* has been projected to spread (Raghavan et al., 2019; Namgyal et al., 2020). The present study investigates the intersection of an invasive ixodid tick and a tick-borne flavivirus, ultimately demonstrating that *H. longicornis* is a competent vector for POWV II under laboratory conditions. At present, it is unknown whether non-viremic transmission of POWV can occur between native tick species (e.g., *Ixodes scapularis*) co-feeding on the same host with *H. longicornis*. If interspecies co-feeding transmission of POWV is possible, then invasive *H. longicornis* populations may increase infection rates in native tick populations, which could increase the risk of human infection. Studies investigating *H. longicornis*’ ability to acquire and transmit POWV *via* co-feeding are underway.

## Data Availability Statement

The original contributions presented in the study are included in the article/**Supplementary Material**. Further inquiries can be directed to the corresponding author.

## Ethics Statement

The animal study was reviewed and approved by University of South Alabama Institutional Animal Care and Use Committee.

## Author Contributions

Conceptualization: MH; Data curation: WR; Formal analysis: WR, EH, IL, MS, MH; Funding acquisition: MH; Methodology: WR, EH, IL, MS, MH; Project administration: MH; Supervision: MH; Validation: WR, EH, IL, MS; Writing (original draft): WR; Writing (review & editing): WR, IL, MS, MH.

## Funding

This study was supported by the University of South Alabama College of Medicine and the National Institute of Allergy and Infectious Diseases, National Institutes of Health (award no. R21AI163693).

## Conflict of Interest

The authors declare that the research was conducted in the absence of any commercial or financial relationships that could be construed as a potential conflict of interest.

## Publisher’s Note

All claims expressed in this article are solely those of the authors and do not necessarily represent those of their affiliated organizations, or those of the publisher, the editors and the reviewers. Any product that may be evaluated in this article, or claim that may be made by its manufacturer, is not guaranteed or endorsed by the publisher.

## Supplementary Material

Supplementary Figure 1 Clinical markers of POWV-disease progression in mice infested with media-injected *H. longicornis*. **(A)** Percent weight change is represented as a line plot relative to the weight at study day 0. **(B)** Clinical disease scores are represented as a line plot with higher cumulative scores representing more severe disease. Cumulative clinical scores were assigned to each mouse daily based on weight loss, appearance, neurological signs of disease, and behavior.

Supplementary Figure 2 Detection of POWV RNA in blood from mice used in tick acquisition feeding experiment. Mice were intraperitoneally injected with POWV II, infested with pathogen-free larval and nymphal *H. longicornis*, then bled at 1 and 3 days post-injection (d.p.i.). The viremia data are presented as means with standard errors of the means. Statistical significance was determined using a two-tailed unpaired t test, **P < 0.01. Limit of detection ~15 gene fragment copies per ng RNA.
