# Supplementary figures and images for: Horizontal and Vertical Transmission of Powassan Virus by the Invasive Asian Longhorned Tick, Haemaphysalis longicornis, Under Laboratory Conditions
Source: Front Cell Infect Microbiol. 2022 Jul 1;12:923914. doi: 10.3389/fcimb.2022.923914 (PMC9283711; doi:10.3389/fcimb.2022.923914)

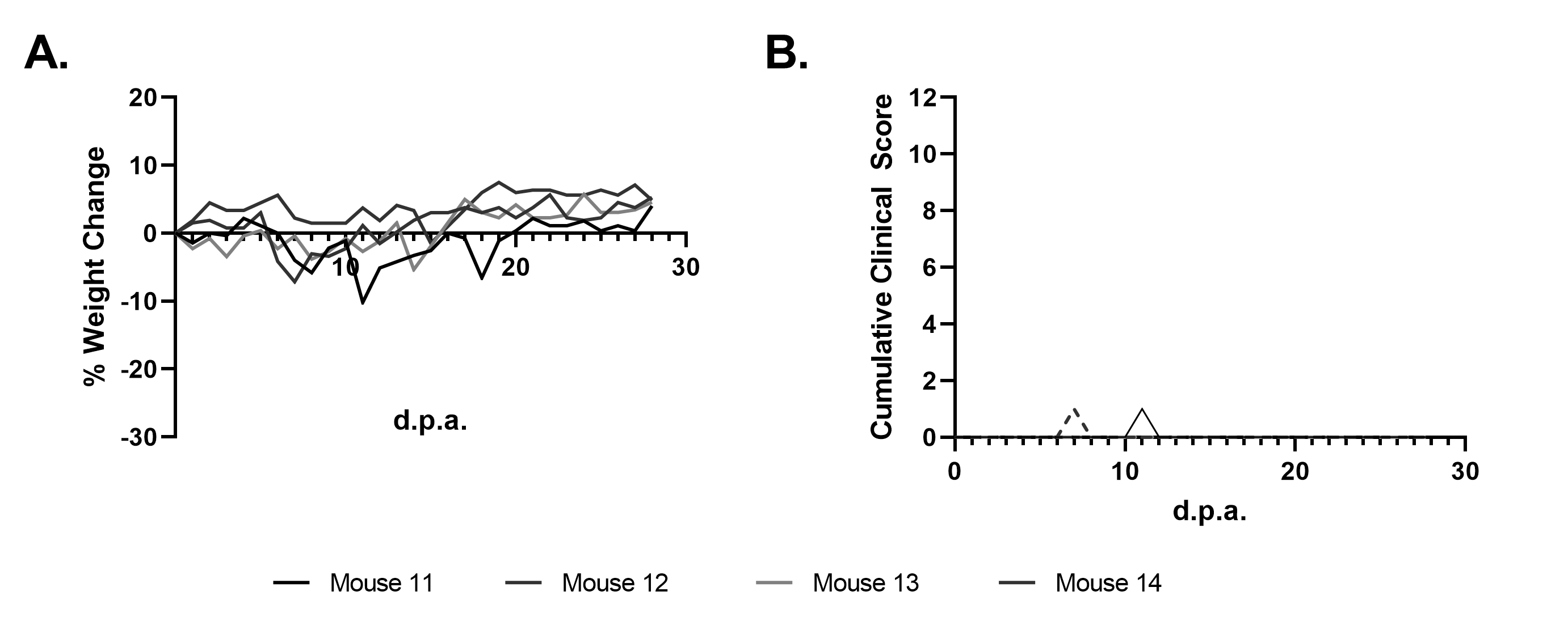

Supplement: Supplementary Figure 1 — Clinical markers of POWV-disease progression in mice infested with media-injected H. longicornis. (A) Percent weight change is represented as a line plot relative to the weight at study day 0. (B) Clinical disease scores are represented as a line plot with higher cumulative scores representing more severe disease. Cumulative clinical scores were assigned to each mouse daily based on weight loss, appearance, neurological signs of disease, and behavior. [file Image_1.tif]

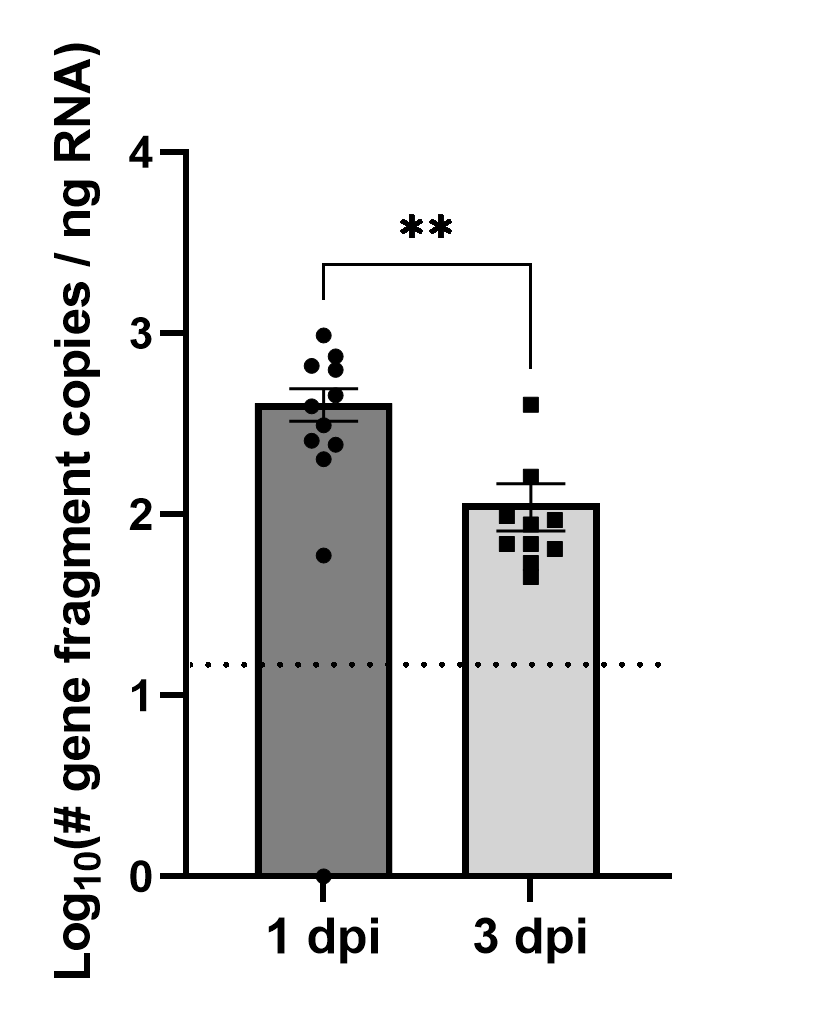

Supplement: Supplementary Figure 2 — Detection of POWV RNA in blood from mice used in tick acquisition feeding experiment. Mice were intraperitoneally injected with POWV II, infested with pathogen-free larval and nymphal H. longicornis, then bled at 1 and 3 days post-injection (d.p.i.). The viremia data are presented as means with standard errors of the means. Statistical significance was determined using a two-tailed unpaired t test, **P < 0.01. Limit of detection ~15 gene fragment copies per ng RNA. [file Image_2.tif]
